# A Bespoke Electronic Health Record for Epilepsy Care (EpiToMe): Development and Qualitative Evaluation

**DOI:** 10.2196/22939

**Published:** 2021-02-12

**Authors:** Shiqiang Tao, Samden Lhatoo, Johnson Hampson, Licong Cui, Guo-Qiang Zhang

**Affiliations:** 1 Department of Neurology The University of Texas Health Science Center at Houston Houston, TX United States; 2 Texas Institute for Restorative Neurotechnologies The University of Texas Health Science Center at Houston Houston, TX United States; 3 School of Biomedical Informatics The University of Texas Health Science Center at Houston Houston, TX United States

**Keywords:** specialty-specific EHR, physician-centered design, clinical workflow, patient care management, clinical care documentation, physician burnout, interoperability

## Abstract

**Background:**

While electronic health records (EHR) bring various benefits to health care, EHR systems are often criticized as cumbersome to use, failing to fulfill the promise of improved health care delivery with little more than a means of meeting regulatory and billing requirements. EHR has also been recognized as one of the contributing factors for physician burnout.

**Objective:**

Specialty-specific EHR systems have been suggested as an alternative approach that can potentially address challenges associated with general-purpose EHRs. We introduce the Epilepsy Tracking and optimized Management engine (EpiToMe), an exemplar bespoke EHR system for epilepsy care. EpiToMe uses an agile, physician-centered development strategy to optimize clinical workflow and patient care documentation. We present the design and implementation of EpiToMe and report the initial feedback on its utility for physician burnout.

**Methods:**

Using collaborative, asynchronous data capturing interfaces anchored to a domain ontology, EpiToMe distributes reporting and documentation workload among technicians, clinical fellows, and attending physicians. Results of documentation are transmitted to the parent EHR to meet patient care requirements with a push of a button. An HL7 (version 2.3) messaging engine exchanges information between EpiToMe and the parent EHR to optimize clinical workflow tasks without redundant data entry. EpiToMe also provides live, interactive patient tracking interfaces to ease the burden of care management.

**Results:**

Since February 2019, 15,417 electroencephalogram reports, 2635 Epilepsy Monitoring Unit daily reports, and 1369 Epilepsy Monitoring Unit phase reports have been completed in EpiToMe for 6593 unique patients. A 10-question survey was completed by 11 (among 16 invited) senior clinical attending physicians. Consensus was found that EpiToMe eased the burden of care documentation for patient management, a contributing factor to physician burnout.

**Conclusions:**

EpiToMe offers an exemplar bespoke EHR system developed using a physician-centered design and latest advancements in information technology. The bespoke approach has the potential to ease the burden of care management in epilepsy. This approach is applicable to other clinical specialties.

## Introduction

### Electronic Health Records

Electronic health records (EHR) have been broadly adopted in the United States in the last 2 decades to improve the quality of health care, increase patient satisfaction, and save health care costs [1-3], as mandated by the Health Information Technology for Economic and Clinical Health (HITECH) Act of 2009 [4-7]. Compared to paper-based medical records, EHR has advantages that include easier access, higher working efficiency, increased patient satisfaction, reduced financial cost, better data exchange and interoperability, and opportunities for secondary use of clinical data for research [8-11].

While EHR brings such benefits to health care, EHR systems are often criticized as cumbersome to use, failing to fulfill the promise of improved health care delivery with little more than a means of meeting regulatory and billing requirements [12]. A recent study inspected the time allocation pattern among over 31 million transactions for 471 physicians from 2011 to 2014 and found that physicians spent progressively more time on “desktop medicine” and less on face-to-face patient care [13]. Another study inspected EHR event logs and showed that primary care physicians spend more than half of their workday interacting with the EHR during and after clinic hours [14].

EHR systems have been recognized as one of the contributing factors for physician burnout [15,16], an increasing health care crisis in the United States [17-20]. Burnout is on the rise and affects all specialties [21]. Studies show that burnt-out doctors are more likely to make medical errors [22], work less efficiently [23], and have higher referral rates [24]. A recent survey of nearly 6880 physicians reported that 1 in 50 planned to leave medicine altogether in the next 2 years, while 1 in 5 planned to reduce clinical hours over the next year [25]. Another study [26] reported that 26% of 1792 physician respondents reported burnout, and 70% of 1631 users reported EHR-related stress. The study also reported that high rates of fatigue among intensive care unit physicians were associated with low EHR efficiency [27].

### Specialty-Specific EHRs

One recent study pointed out that different specialties had different unique requirements, and this difference should be reflected in EHR design and implementation [28]. Specialty-specific or bespoke EHR is a promising approach to overcoming the limitations of general-purpose EHR and mitigating physician burnout. A bespoke EHR is an EHR custom designed to meet the unique needs of providers in a specific specialty or care setting. Bespoke EHR can prevent clinicians from spending a significant portion of their workday sifting through large amounts of clinical data for the specific data elements they need. In another recent study [29], it was reported that a clinic-focused Sprint process can optimize EHR efficiency and have positive effects on physician burnout. Specialty-specific EHR improvement is one major intervention during the Sprint process. In general, specialty-specific EHR can better achieve the level of optimization and workflow management expected by physicians [30], although approaches based on EHR customization have limitations in what is achievable compared to a bespoke design built from the ground up. Standalone, specialty-specific EHRs have been around for a number of years in such areas such as emergency medicine, ophthalmology, and dermatology. However, broader adoption of such a specialty-specific approach faces challenges in interoperability between different EHR systems, capturing standardized structured data for documenting care, and supporting the data-readiness needs to drive a learning health system.

### EpiToMe: A Bespoke EHR

We developed EpiToMe (an Epilepsy Tracking and optimization Management engine), a bespoke EHR system customized for epilepsy care created de novo. EpiToMe has evolved from and integrates clinical applications we have developed over the last decade [31-34]. EpiToMe provides patient data capture functions for electroencephalogram (EEG) reporting, daily reporting, and phase reporting for Epilepsy Monitoring Units (EMUs). It uses domain-specific epilepsy and seizure ontology (EpSO) [35] to (1) support structured entry of multimodal epilepsy data, (2) proactively ensure the quality of data through the use of ontology terms in faceted systems, (3) organize and index patient information for subsequent analytical queries and secondary use, and (4) seamlessly make just-in-time and right-in-context communications with the parent EHR. EpiToMe was developed following web interface–driven development [33], an agile software development methodology, in close collaboration with physicians. EpiToMe has a built-in physician dashboard optimized for physician needs to perform tasks without switching systems or changing navigation interfaces. EpiToMe's data entry pipeline allows other clinicians in the team such as EEG technicians and clinical fellows to take responsibility for appropriate patient data documentation work. EpiToMe also provides a tracker to provide an overview of patient status in the clinical care workflow.

## Methods

### Physician-Centered Design and Interface-Driven Development

Physician interfaces play a critical role in EHR systems and are the most important factor affecting usability and clinical efficiency [12,29]. However, in the history of EHR development, physicians have rarely had a major role in deciding how an EHR interface should be built. Modern EHR systems (Allscripts, EPIC, and Cerner) offer physicians some opportunity to provide document templates, but physicians often neither have the expertise to optimize such templates nor do they have the flexibility to maintain or update these templates as needed.

**Figure 1:**
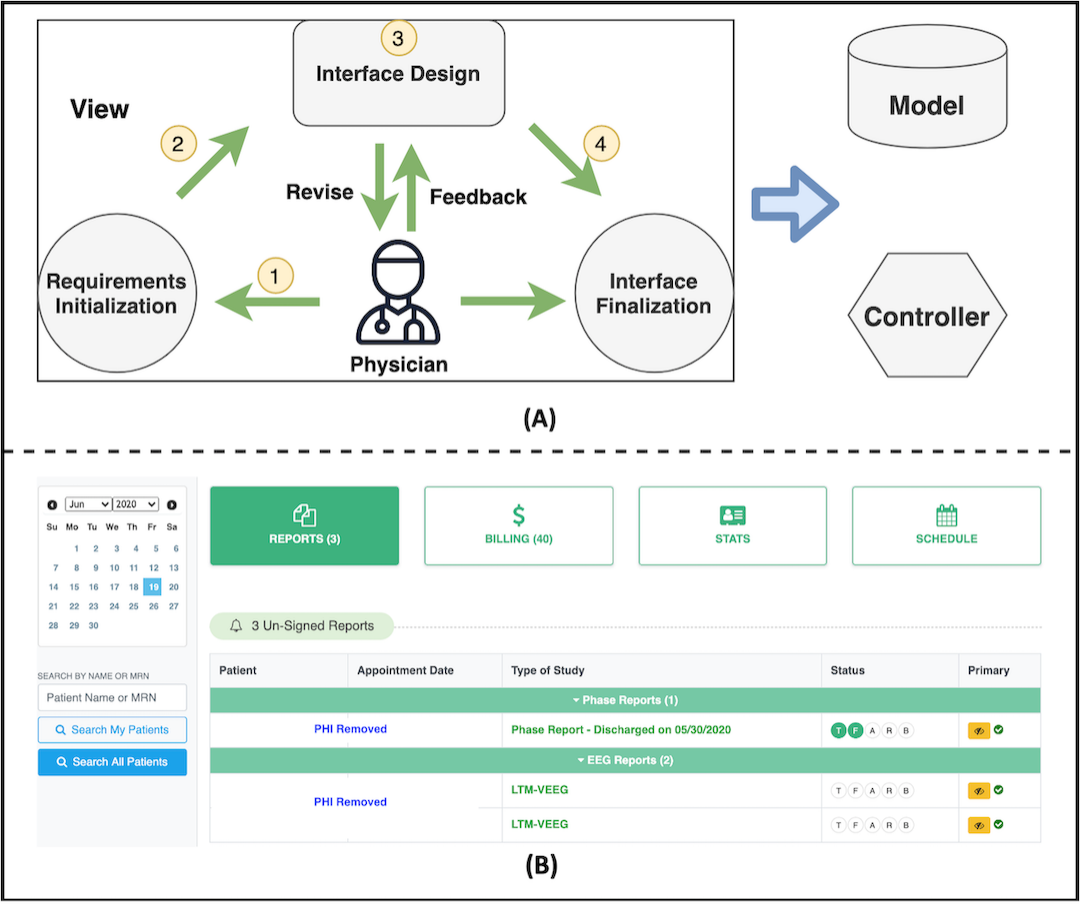
(A) Steps involved in our physician-centered design and interface-driven development process; (B) Exemplar physician dashboard resulting from our physician-centered design. LTM-VEEG: Long-term Video Electroencephalogram; PHI: Protected Health Information.

In EpiToMe, we address this problem using an agile, physician-centered design and interface-driven development during all stages of the development process from inception. As depicted in Figure 1A, EpiToMe follows the classic model-view-controller architectural pattern. We use user interfaces to drive the development of data models and controllers. Our interface design process consists of 4 steps with physicians in the loop: (1) The process starts with physicians’ initial requirements; (2) then, informaticians complete the next iteration of the interface prototype incorporating such requirements; (3) physicians give feedback about the prototype and working interfaces and suggest revisions to be made in the future iterations; and (4) step 3 continues iteratively until the design is accepted and finalized by physicians and a testing or production version is deployed.

With this physician-centered design and interface-driven development method, EpiToMe ensures that the interfaces have the look, feel, and functionality desired by physicians, improving user satisfaction and optimizing clinical efficiency (eg, Figure 1B).

### Functional Architecture

**Figure 2:**
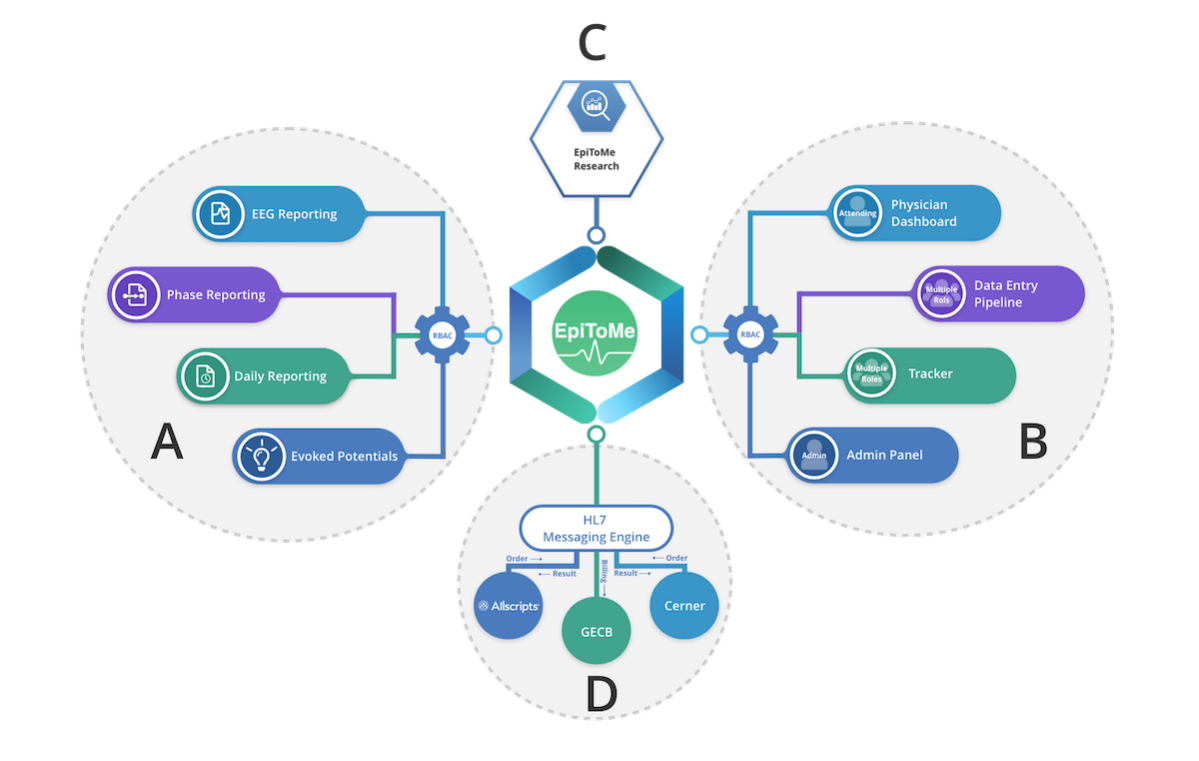
EpiToMe's functional architecture with 4 major functional areas: (A) data capture interfaces for clinical reports, (B) data and analytical dashboards, (C) clinical research query interface, (D) HL7 messaging engine for communication with background electronic health record systems (Allscripts, Cerner, and EPIC). Role-based access control can be configured to manage who gets which levels of access to what information, as defined by their clinical roles. EEG: electroencephalogram.

Figure 2 shows the functional architecture design of EpiToMe, consisting of 4 major areas. Figure 2A shows the data capture interfaces for clinical reports. Currently, 4 types of clinical reports are built in EpiToMe: EEG Reporting, Phase Reporting, Daily Reporting, and Evoked Potentials. These interfaces capture essential diagnostic information for epilepsy care, which are then seamlessly pushed to the general EHR (see Figure 2D). Figure 2B shows the data dashboards. The Physician Dashboard allows a physician to track outstanding reports, file for billing, monitor statistics of activity in a given time interval, and review the service schedule. The tracker is an interactive, real-time interface displaying each patient’s status in the entire epilepsy care workflow, from admission and discharge from EMU to postoperative evaluations. Figure 2C shows the clinical research query interface. This is a faceted interface for ad-hoc, on-the-fly identification and construction of epilepsy patient subgroups for research. In EpiToMe, all patient information including demographics, diagnoses, epilepsy-related clinical characteristics, and medications are indexed to make such information queriable and exportable. Figure 2D shows the interoperability through an HL7 messaging engine. HL7 is a widely used protocol for the transfer of clinical and administrative data among EHR systems [36]. EpiToMe implemented 3 primary HL7 standard message types: orders, results, and charges for epilepsy care. The EpiToMe HL7 messaging engine allows it to seamlessly communicate with the general EHR. EpiToMe receives orders from the general EHR and sends back the completed reports and billing messages.

### Collaborative, Asynchronous Care Documentation

Clinical care comprises collaborative teamwork from different clinical stakeholders; patient data documentation should follow suit. We designed the EpiToMe data entry pipeline to be automatically triggered when an order is placed in the general EHR. An order message containing patient demographics and order details is sent to EpiToMe. Based on the message, EpiToMe will create a new report document for the order and notify EEG technicians that a patient report is waiting to be handled. Next, an EEG technician will perform the EEG recording on patients and document the EEG specifications in EpiToMe. EpiToMe will mark the report as “technician completed” and pass it to clinical fellows who read the EEG recordings and enter their interpretations in the report. After fellows complete their data entries, the report will appear in the physician dashboard, and the clinical attending physicians will take it over, review it, and finalize it.

### Role-Based Access Control for Collaborative Data Entry

EpiToMe applies a role-based access control (RBAC) method to manage users’ access to data and interfaces. RBAC is a popular framework for implementing the security policy of an organization’s enterprise information system. In RBAC, permissions are associated with roles, and roles are assigned to users. We designed EpiToMe’s RBAC so that every user is assigned one or multiple roles, and each role defines what actions are allowed to perform within the system. To fully reflect the physician-centered interface design, RBAC needs to be implemented not only at the data access level but also at the interface level. Our RBAC method ensures that users can focus on the responsibility corresponding to individual clinical roles, thereby improving efficiency.

### Ontology-Driven Data Capture

EpiToMe uses EpSO to provide a standard vocabulary and guide the data entry for all clinicians. EpSO provides more than 600 terms, which include epileptic diagnoses, epilepsy semiologies, epileptogenic zones, lateralizing signs, EEG activities, and etiologies. We designed a dedicated widget in the style of multilevel dropdowns for clinicians to enter patient data. This widget supports hierarchical “hover to expand” operation, allowing users to locate the desired terms efficiently. With the ontology-guided data entry method, users select data items instead of typing them, which prevents possible common data quality issues such as typos and inconsistencies.

### Interoperability Using HL7 Information Exchange

EpiToMe handles the epilepsy-related orders from multiple locations. Two different EHRs are used in these locations: Allscripts (EHR of University of Texas [UT] physicians) and Cerner (EHR of Memorial Hermann Health System). We designed an HL7 engine that can consume HL7 messages from multiple EHR vendors.

**Figure 3:**
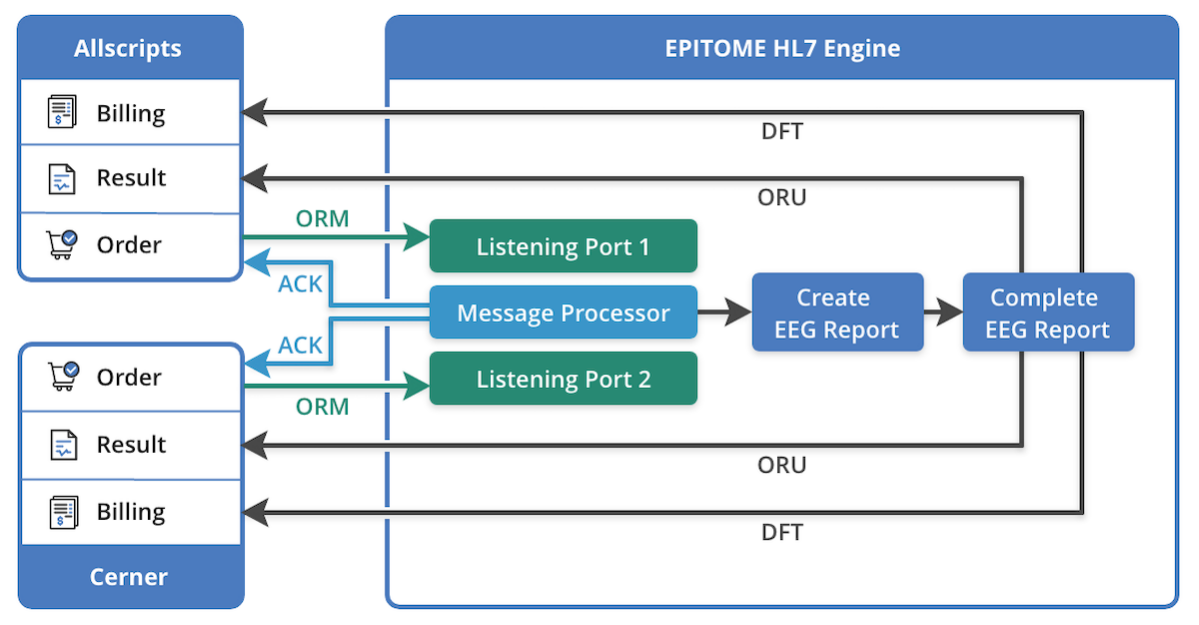
HL7 messaging interface between EpiToMe and the parent electronic health record systems. ACK: Acknowledgement; DFT: Detail Financial Transaction; EEG: electroencephalogram; ORM: Order Entry Message; ORU: Order Result.

Figure 3 shows the architecture of the HL7 engine. HL7 messages are used to transfer electronic data between disparate health care systems. Each HL7 message sends information about a particular event such as a patient admission or a lab test order. Three primary HL7 standard message types are handled in EpiToMe: orders (ORM), results (ORU), and charges (DFT). ORM messages contain patient demographic information and order-related data. ORU is usually in response to an order and provides clinical observations. DFT is used to send billing information. EpiToMe receives order messages from Allscripts and Cerner and creates patient reports with the embedded information in the order messages. EpiToMe confirms the order messages by sending back acknowledgement (ACK) messages. Physicians complete these reports in EpiToMe and then send them back to the original EHR system with ORU messages. After the results are accepted, physicians can continue to file billing messages using EpiToMe. Our HL7 engine design allows EpiToMe to seamlessly communicate with the parent EHR systems.

### Assessment Survey Design

To assess the EpiToMe’s effectiveness in improving the user experience with patient documentation and reducing physician burnout, we designed an online survey administered within EpiToMe. Physicians users of the EpiToMe system were invited to participate in the survey. The survey (Table 1) consists of 10 questions addressing the common concerns about physicians’ dissatisfaction with general EHR systems including the length of time spent on patient documentation, face-to-face interaction opportunities with patients, and catching up with care documentation in off-work time [13-16]. The specific EHR systems that are compared with EpiToMe in this survey are Allscripts and Cerner. Our design of the questionnaire was also informed by the Maslach Burnout Inventory Manual [37]. Questions 1-8 were designed to have Likert rating scales from 1 to 5, representing strongly disagree (1), disagree (2), neutral (3), agree (4), and strongly agree (5). Questions 9 and 10 are open ended to solicit input in free-text form. Specifically, question 9 asks physicians to enter which aspects of EpiToMe help with addressing physician burnout. Question 10 solicits the features physicians would like to see implemented in a future version of EpiToMe.

**Table 1:** The 10 survey questions and their answer options.

Question	Answer type
1. My overall workflow is less frustrating with EpiToMe compared to before.	1-5 rating scale
2. Completing patient reports with EpiToMe is easier and more intuitive than with EHR.	1-5 rating scale
3. I spend less amount of time billing using EpiToMe compared to using EHR.	1-5 rating scale
4. I spend less after-work time catching up with reports or billing using EpiToMe.	1-5 rating scale
5. EpiToMe allows me to spend more time on direct patient care.	1-5 rating scale
6. My oversight of the patient journey from the clinic to epilepsy surgery is better with EpiToMe.	1-5 rating scale
7. The dashboard in EpiToMe helps me to know my task list and complete it appropriately.	1-5 rating scale
8. For epilepsy reporting and billing, I would prefer using EpiToMe compared to EHR.	1-5 rating scale
9. In my opinion, the aspects of EpiToMe which help me address physician burnout are:	Free text
10. In my opinion, the additional features that I would like to see implemented in EpiToMe are:	Free text

## Results

### Physician Dashboard

#### Overview

With the physician-centered design, we created a physician dashboard—an integrated interface specially designed for epilepsy care providers. As shown in Figure 1B, the physician dashboard consists of 4 tabs: reports, billing, statistics, and schedule. EpiToMe directly leads physicians to this dashboard reflecting the present date status when they log in, where they can manage all the day-to-day tasks by selecting a specific day on the calendar without the need to navigate between different web pages or switch to different systems.

#### Reports

The default function tab is “Reports.” The number of outstanding reports to be completed is displayed in the bracket following the tab title. Physicians can review and complete reports here and send the completed reports back to the parent EHR systems with one button click.

#### Billing

After the reports are accepted by the parent EHR, physicians can continue to work on the billing. As a bespoke EHR system, EpiToMe automatically pulls all billing-related information and displays it in a user-friendly style in the billing interface. Physicians can file billing for a report with 3 to 4 clicks. In contrast, it takes more than 24 clicks on multiple pages to complete the same task in the billing interface of the general-purpose EHR system.

#### Statistics

The “Statistics” tab provides an overview of reports completed, documented, and billed by physicians, including the number of reports by month and type of study.

#### Schedule

In the “Schedule” tab, physicians can review their service schedules for the whole year. It allows a physician to send requests to another physician to switch schedules, which was a significant challenge in the previous schedule management system. The implementation of the schedule functionality also allows EpiToMe to automatically link reports to their corresponding physicians.

### Usage Summary

EpiToMe creates interfaces for 4 types of reports for epilepsy care: EEG report, EMU phase report, EMU daily report, and evoked potentials. Table 2 shows the statistics for these reports. The EEG report is the first type of reporting function for production use in EpiToMe. By September 21, 2020, clinicians had completed 15,417 EEG reports in EpiToMe since its first launch on February 18, 2019. The EMU phase report is the second reporting interface in production use since July 1, 2019. A total of 1369 EMU phase reports have been completed in EpiToMe. EpiToMe also has documented 2635 EMU daily reports since its production date of November 15, 2019. The evoked potentials reporting function is under testing. Combining these reports, EpiToMe has documented 19,421 reports for 6593 unique patients.

**Table 2:** EpiToMe report statistics.

Report type	Status	Status date	Number of reports (N=19,421)	Number of patients (N=6593)
EEG^a^ report	Production	02/18/2019	15,417	6382
EMU^b^ phase report	Production	07/01/2019	1369	1053
EMU daily report	Production	11/15/2019	2635	312
Evoked potentials	Test	N/A^c^	0	0

^a^EEG: electroencephalogram.

^b^EMU: Epilepsy Monitoring Unit.

^c^N/A: not applicable.

### Patient Tracking Interface

**Figure 4:**
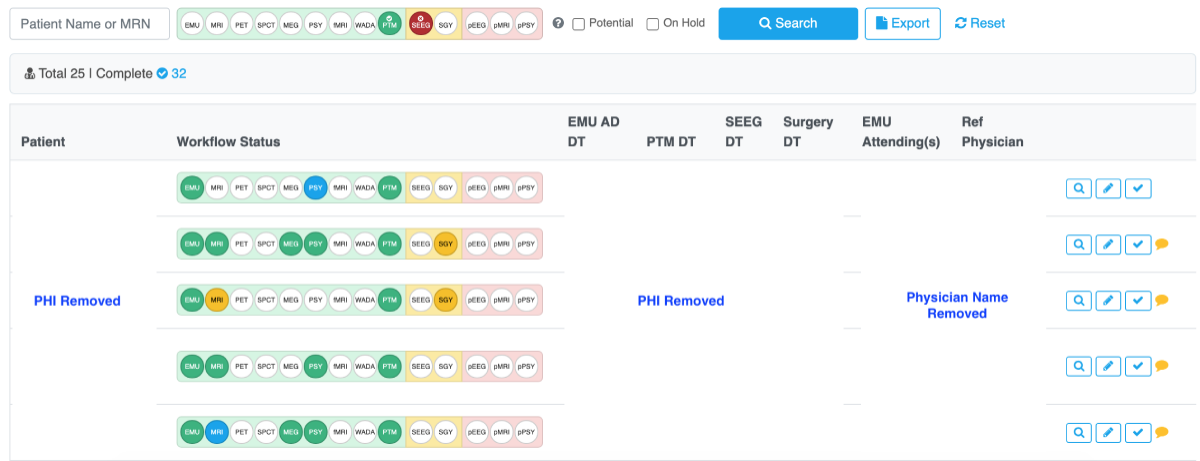
Tracker of epilepsy surgery candidates. AD: admission; DT: date; EMU: Epilepsy Monitoring Unit; MRN: medical record number; PHI: protected health information; PTM: patient management; Ref: referring; SEEG: stereoelectroencephalography.

Epilepsy care is a complex process that requires the collaboration of multiple clinical teams, including neurologists, radiologists, neuropsychologists, and neurosurgeons, especially for patients who are not responsive to seizure-control medications and treated as surgical candidates. It is critical to keep track of the patient status in the clinical workflow from 2 perspectives: (1) Keep different clinical teams on the same page, and (2) identify and resolve the bottleneck in the workflow. EpiToMe creates a patient tracking interface called a tracker. As displayed in Figure 4, the tracker records 14 possible steps of the patient journey in epilepsy care, which include admission to EMU, discharge from EMU, positron emission tomography scan, Ictal single-photon emission computed tomography, magnetoencephalography, neuropsychology, functional magnetic resonance imaging, Wada, patient management, stereoelectroencephalography, surgery, postoperative EEG, postoperative magnetic resonance imaging, and postoperative neuropsychology. Each circle represents a clinical step. Color codes are used to indicate the status of completion of each step: Blue indicates a test is ordered, yellow means a procedure is scheduled, and green shows that a step is complete.

The search functionality in the tracker provides a search mechanism for users to quickly find a patient or a cohort of patients. In addition to searching by name or medical record number, users can search patients by the completion status of each step and combine statuses to get results. In the search template, red status means a step has not started yet. Figure 4 shows an example of searching patients who have had patient management conferences but pending stereoelectroencephalography.

The tracker also provides comments functionality for users with the role of a nurse navigator, which is the specific user role responsible for updating the tracker in EpiToMe. The nurse navigator will enter comments once a bottleneck is identified and notify all related clinical teams to keep them alert and encourage teamwork to resolve the bottleneck.

### Collaborative, Asynchronous, Data Entry Pipeline

**Figure 5:**
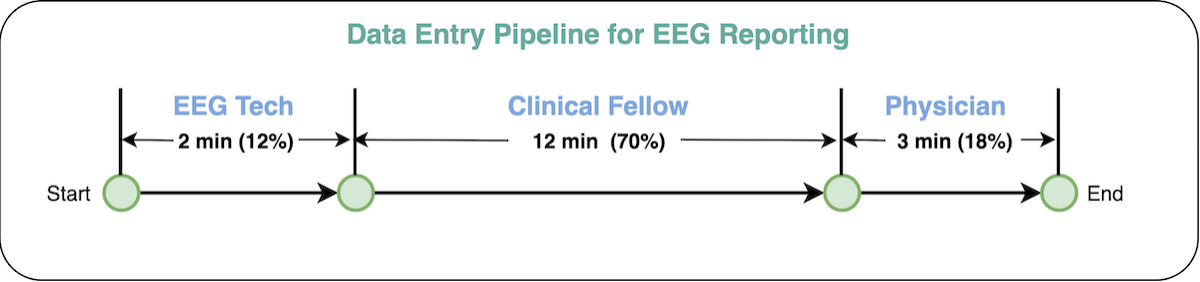
Collaborative data entry pipeline which reduces physician burden with patient documentation. EEG: electroencephalogram.

With the physician-desired interface and RBAC in place, EpiToMe implements a collaborative data entry pipe to improve clinical efficiency and distribute patient documentation workload. Figure 5 shows the usage statistics of the data entry pipeline with EEG reporting. In this pipeline, the physician is not the only role that completes patient documentation. Instead, the result of inspecting the user activity logs shows that the physician’s average time spent on the EEG reporting only occupies 18% of the total time of all clinical roles.

### Survey Results

**Figure 6:**
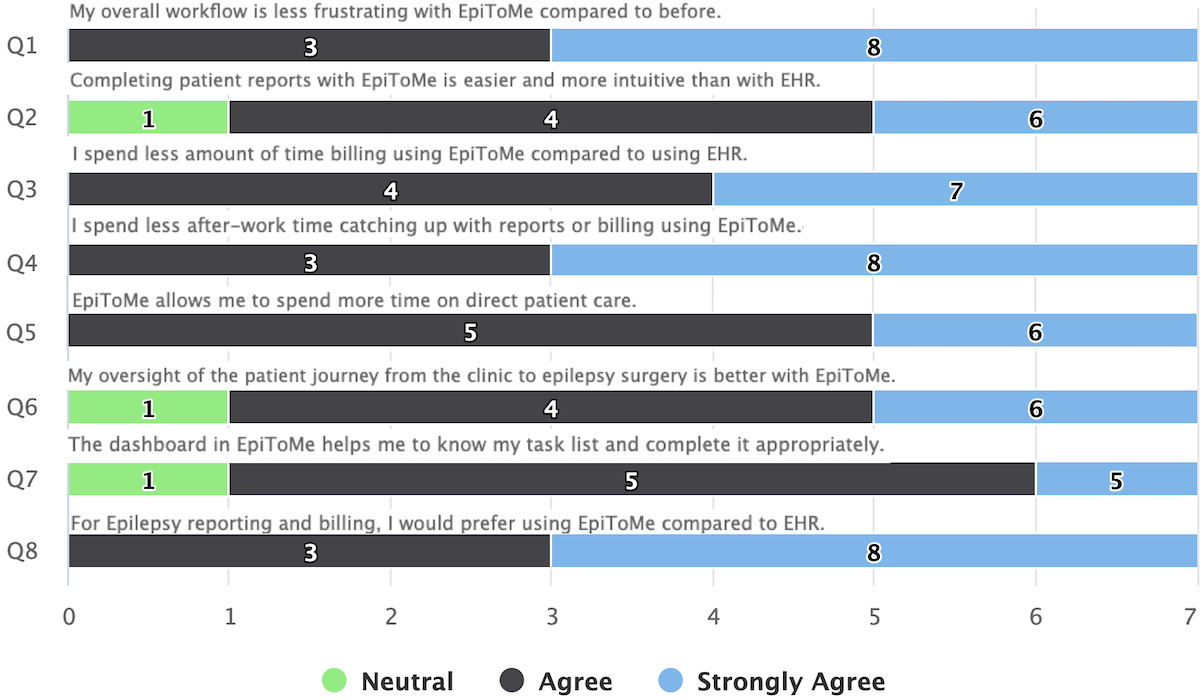
Responses for survey questions 1-8. EHR: electronic health records.

Of the 16 survey invitations sent to physicians, we received 11 completed responses. Answers to each of the questions 1-8 are displayed in Figure 6. In general, physicians favored EpiToMe for patient reporting and billing compared to the general EHR. All physicians agreed that the workflow with EpiToMe was less frustrating, and most (8/11, 73%) strongly agreed on this. Most physicians (10/11, 91%) thought EpiToMe made it easier to complete patient reports, and all physicians agreed that billing with EpiToMe took less time than previously using the general EHR systems. As a result, all physicians reported in question 4 that they spent less off-work time catching up with reports or billing, which is considered a significant factor for physician burnout. All physicians agreed that EpiToMe allowed them to spend more time on direct patient care, and most physicians (10/11, 91%) thought EpiToMe helped them better manage the patient journey and their task list.

For question 9, physicians described the aspects of EpiToMe that helped them address physician burnout. One respondent said: “Intuitive, fast, accurate, comprehensive interactions; everything makes sense; very little redundancy.” Another respondent pointed out “ease of reporting and billing” as well as “no need of separate data sets (eg, personal spreadsheets, email lists) to keep track of patients and testing.” Another physician endorsed the integrating role of EpiToMe as “It reduces the number of places (different EHR systems) I need to be working on simultaneously—the integration between reporting and billing reduces the amount of time spent on non-patient–care related work.” One physician also appreciated the collaborative data entry pipeline: “better time management and easier/more efficient interface with the fellows in terms of report writing and billing on time.”

In response to question 10, physicians proposed many constructive suggestions that can serve as future directions for EpiToMe. These include “more EHR interfaces,” “outpatient module,” and being more user-friendly such as “being able to have multiple sections within the report open while editing the report.”

## Discussion

### Physician Feedback

With a specialty-specific, physician-centered interface design, EpiToMe can improve overall clinical efficiency. For example, for EEG reporting, the average time delay (from the completion of EEG recording to the finalization of the EEG reporting) to complete an EEG report is 14 hours and 30 minutes for 98% of EEG reports. Patients obtain their EEG reports within the same day of visit rather than a couple of days or weeks later using the previous general-purpose EHR. Survey feedback by clinical attending physicians showed significant preference for using EpiToMe to perform reporting and billing tasks compared with general-purpose EHR systems. Within the 88 answers from 11 senior clinical attending physicians, 85 (96.6%) indicated that EpiToMe is better than the general EHR on specific tasks, with only 3 neutral answers.

### Multisite Deployment

EpiToMe is a multisite system supporting interoperability with multiple types of EHR systems. Currently, EpiToMe has been deployed at 4 clinical centers including UT Physicians Clinic, Memorial Hermann Texas Medical Center, Memorial Hermann The Woodlands Medical Center, and Memorial Hermann Cypress Hospital. Within these medical centers, UT Physicians Clinic uses Allscripts as their general-purpose EHR system, while others use Cerner. We are making EpiToMe also interoperable with Epic as UT Physicians Clinic transitions from Allscripts to Epic.

### Interoperability

HL7 is a widely used standard for data exchange in clinical information systems. Implementation of an HL7 messaging engine enables EpiToMe to interoperate and exchange information with general-purpose EHR systems, resulting in significantly reduced or eliminated redundant data entry. Our HL7 messaging engine also makes EpiToMe scalable: New clinical centers can be added in EpiToMe as long as their EHR platforms offer service to support HL7 communication standards.

### Generalizability

EpiToMe is not a replacement for general-purpose EHR. Instead, it is complementary to existing general EHR solutions. EpiToMe relies on the availability of the parent EHR to admit and transmit patient demographic information and epilepsy-related orders, which triggers the corresponding, additional data capture process in EpiToMe. Although EpiToMe is optimized for epilepsy care, our methodology, design principle, system architecture, and interface elements are generally applicable. In fact, we are applying a similar approach in UTHealth Neurosciences service lines to derive similar benefits for other clinical specialties.

### Survey Limitations

Our physician survey study is preliminary, as this is not the primary focus of the paper. Limitations of this survey include the small sample of survey participants and the lack of complete independence of survey participants and the informatics development team. Such limitations may present hidden bias in survey results, and larger-scale, anonymous surveys are the preferred approach for feedback. However, production-level deployment and long-term operation of interoperable bespoke EHRs implemented using physician-centered design and the latest information technology for clinical specialties are uncommon. Therefore, timely assessment of such bespoke EHRs, even on a smaller scale and with limitations, provides valuable and much-needed operational feedback to inform hospitals’ adoption strategies. Surveys should also accommodate a strategy tailored to the tremendous effort and longer development cycle needed in designing, deploying, and operationalizing such systems in real-world clinical practice and patient care settings. After a few years of operation, a more systematic, comparative activity log analysis would provide more objective insight about where our bespoke approach made the most impact and where further enhancements may be needed.

### Conclusions

Working closely with physicians, we used an interface-driven development approach to create EpiToMe de novo, to embody physician preferences and optimize clinical workflow for epilepsy care while ensuring interoperability with the parent EHR. EpiToMe offers an exemplar pathway to mitigate physician burnout and improve the quality and productivity of care by combining physician-centered design with the latest advances in information technology in a bespoke EHR system.

## Abbreviations

EEG: electroencephalogram
EHR: electronic health records
EMU: Epilepsy Monitoring Unit
EpSO: epilepsy and seizure ontology
HITECH: Health Information Technology for Economic and Clinical Health
RBAC: role-based access control
UT: University of Texas
